# Nucleus-wide analysis of coherent RNA pol II movement in the context of chromatin dynamics in living cancer cells

**DOI:** 10.1080/19491034.2022.2157133

**Published:** 2022-12-13

**Authors:** Haitham A. Shaban

**Affiliations:** a Agora Cancer Research Center, Lausanne, Switzerland; b Precision Oncology Center, Department of Oncology, Lausanne University Hospital, Lausanne, Switzerland; c Spectroscopy Department, Physics Division, National Research Centre, Cairo, Egypt

**Keywords:** DFCC, RNA Pol II live imaging, Quantitative analysis, Chromatin imaging, Transcription regulation

## Abstract

Activation of transcription results in coordinated movement of chromatin over a range of micrometers. To investigate how transcriptional regulation affects the mobility of RNA Pol II molecules and whether this movement response depends on the coordinated movement of chromatin, we used our Dense Flow reConstruction and Correlation (DFCC) method. Using DFCC, we studies the nucleus-wide coherent movements of RNA Pol II in the context of DNA in humancancer cells. This study showed the dependance of coherent movements of RNA Pol II molecules (above 1 µm) on transcriptional activity. Here, we share the dataset of this study, includes nucleus-wide live imaging and analysis of DNA and RNA polymerase II in different transcription states, and the code for teh analysis. Our dataset may provide researchers interested in the long-range organization of chromatin in living cell images with the ability to link the structural genomic compartment to dynamic information. .

## Introduction

In eukaryotes, gene transcription by RNA Pol II critically depends on the spatiotemporal structure of chromatin, to guarantee that proteins exactly arrive at the appropriate positions on the genome to ensure optimal gene expression [1,2]. Transcription is an intricate process that requires multiple players to coordinate with chromatin to maintain cellular activity [3]. Transcription by RNA polymerase II (Pol II) takes place with various movements and speeds, which plays a critical role in controlling gene expression [4]. The interaction between transcription players and chromatin occurs in multiple steps that make their coordination during transcription an extremely dynamic process [5,6]. These transcription players enable transcription initiation and elongation, tightly bind DNA, and limit nucleus-wide chromatin diffusion [7,8].

The dynamic properties of both RNA Pol II and chromatin have been highlighted by improvements in live-cell imaging and labeling technologies [9]. At the single gene level, single-particle tracking technology has been used to visualize and measure individual copies of a single gene and the transcriptional output during transcription stages [6,10]. Using the high-resolution diffusion mapping (Hi-D) technique, RNA Pol II movements in single living cells were also studied at high resolution throughout the nucleus [11,12]. The physical coupling between RNA Pol II and chromatin has recently been demonstrated by super-resolution imaging studies in fixed cells, without the dynamic information of RNA Pol II [13,14]. To overcome this limitation, we introduced the Dense Flow reConstruction and Correlation (DFCC) method [15]. DFCC accurately quantifies the correlation of nuclear macromolecule movements with subpixel sensitivity at the level of a whole nucleus [16,17].

Recently, we applied DFCC to study how transcription regulation affects the mobility of RNA Pol II molecules and whether this response relies on the coordinated movement of chromatin [12]. Generally, the data available for live-cell imaging of chromatin and RNA pol II are mostly from single-particle tracking or low-resolution imaging studies. Moreover, other studies focused only on DNA or RNA pol II, which made it difficult to study the dynamic interaction of chromatin with its environment and the long-range organization of macromolecules in the nucleus. Therefore, we believe that our dataset may provide a basis for researchers interested in the long-rangeorganization of chromatin a foundation to link the structural genomic compartment with dynamic information. Polymer biophysicists studying the kinetics of macromolecules throughout the nucleus can also compare the simulated chromatin and/or the interaction between chromatin and transcription factories with experimental results [18–20]. In addition, the causal relationship between the DNA and RNA Pol II dynamic properties can be inferred [21]. The shared data relate to living nucleus-wide imaging of DNA and RNA polymerase II in different transcription states.

## Materials and methods

### Cell culture

A human U2OS osteosarcoma cell line stably expressing RNA (Ribonucleic Acid) Polymerase II fused with Dendra2 (photoswitchable fluorescent protein Dendra) was used for our study. Cells were cultured in Dulbecco’s modified Eagle medium with phenol red-free (Sigma-Aldrich) supplied with 10% fetal bovine serum (to enable cell growth), 1 mM sodium pyruvate (Sigma-Aldrich), Glutamax contains 50 μg/ml gentamicin (SigmaAldrich), and G418 0.5 mg/ml (Sigma-Aldrich). Cells were cultivated at 37°C with 5% CO2. On 35-mm petri dishes with a #1.5 coverslip-like bottom (-Dish, Ibidi, Biovalley), cells were plated for 24 hours at a density of around 105 cells per dish.

#### DNA staining

For DNA staining, cells were labeled using the SiR-DNA (SiR-Hoechst) kit (Spirochrome AG). SiR-DNA is a far-red fluorophore with high specificity for the DNA minor groove. One mM stock solution was made by dissolving the contents of the SiR-DNA vial in 50 µl of anhydrous dimethyl sulfoxide. This solution was stored at −20 degree Celsius. We diluted the stock solution in a cell culture medium to a concentration of 2 μM and vortexed briefly before labeling. The culture medium was changed to a medium containing SiR-fluorophores on the day of imaging and incubated at 37°C for about 1 hour. Before the imaging, the medium was replaced with L-15 medium (Liebovitz’s, Gibco). For live viewing, cells were mounted on the microscope custom-built 37◦C microscope incubator.

#### Cell starvation, and treatment (transcription inhibition)

### Cell starvation and stimulation

To investigate DNA and RNA Pol II in the condition of an almost non-transcribing state, cells were grown in a serum-free medium. Serum removal arrests cells in G0, and because of the short stimulation with serum, cells are expected to be in the G1 phase of the cell cycle. For stimulation, 10% fetal bovine serum was supplemented to the medium for 15 min. Before imagining it, the cells were mounted in the L-15 medium containing all medium supplements needed for live cell growth both for serum-starved and stimulated conditions.

### Flow cytometry analysis

To compare serum-starved, serum-stimulated, and proliferative cells to the level of RNA in these conditions. The U2OS cells were trypsinized, collected into phosphate-buffer saline at a concentration of 2 106 cells/ml, and then added to a fixative of ice-cold 70% ethanol to represent each condition (with serum, without serum for 24 h, and without serum during 24 h + 15 min serum). After being fixed for at least 2 hours, the cells were washed twice in a FACS buffer (PBS with 2% (v/v) heat-inactivated, sterile-filtered fetal bovine serum and 1 mM EDTA added). The cells were then incubated at 37°C for 45 min in the dark with 2 g/ml of Hoechst 33,324 diluted in FACS buffer, followed by 30 min in the dark with 4 g/ml of Pyronin Y diluted in FACS buffer. Prior to being examined with an LSR II flow cytometer (BD Biosciences), the samples were stored in the dark at 4°C. Hoechst 33,342 and Pyronin Y staining were assessed with UV (350 nm) and yellow green (561 nm) lasers, respectively. Pyronin Y and Hoechst 33,342 were used to measure the amounts of RNA and DNA, respectively. Cells classified as being in the G0 phase were defined as the population with 2 N DNA content and RNA content lower than those of cells classified as being in the G1 phase. To determine if mRNA production increased following serum addition, the medians of Pyronin Y staining for each condition were compared.

### Transcription blocking

To test the RNA Pol II motions under the blocking transcriptional condition, we have added treatment, 5,6-Dichloro-1-β-D-ribofuranosylbenzimidazole (DRB) is added to our experiment, to study how the spatial correlation of RNA Pol II responds to the inhibition of transcription elongation (the stage when RNA strands get longer). DRB (53–85-0) is a classic inhibitor of transcription by RNA polymerase II. It interrupts cyclin-dependent kinase 9 (CDK9) phosphorylation causing pausing transcription elongation. DRB was added to the cell in normal growing conditions, where cells were treated by adding 100 μM DRB (Sigma-Aldrich) to the L-15 imaging medium that contained 10% fetal bovine serum.

### Cell fixation

To fix our cell, the U2OS cells were gently washed with a pre-warmed (37°C) phosphate buffered saline (PBS), then the cells were preserved in 4% (vol/vol) paraformaldehyde in PBS for 10–20 min at room temperature. Before imaging, the cells were washed with PBS (3 times, 5 min each).

### Microscopy and image capture

DNA videos were captured using a Leica Microsystems DMI8 inverted automated microscope with a confocal spinning disc unit (CSU-X1-M1N, Yokogawa). For excitation, an integrated laser engine (ILE 400, Andor) with a selected wavelength of 647 nm (140 mW) was used. Oil immersion objectives (Leica HCXPL-APO 100x/1.4 NA) were used to image the samples.

A single-band bandpass filter (FF01-650/13-25, Semrock, Inc.) was used to filter the SiR-Hoechst fluorescence emission. An image series of 150 frames with an exposure time of 200 ms (5 fps) were captured with Metamorph software (Molecular Devices) and detected with sCMOS cameras (ORCA-Flash4.0 V2) and (1 1 binning). All videos for all conditions were recorded at 37°C in a humid chamber. The sample pixel size of 65 nm.

An image series of 150 frames for RNA Pol II (RPB1-Dendra2) were acquired using a Nipkow-disk confocal system (Revolution, Andor) with a confocal spinning disc unit (CSU22, Yokogawa) with an exposure time of 200 ms. RPB1-Dendra2 was excited at 488 nm using a diode-pumped solid-state laser with a single laser line (25 mW; Coherent). The images were acquired using an oil immersion objective (100×, Plan Apo 1.42, Nikon). An emission filter (ET525/30-25, Semrock) was used to filter the data. The fluorescent emission was detected using a Charge Coupled Device (EMCCD) (iXon Ultra 888), with an 88 nm sample pixel size. IQ software is used to control the system (Andor).

## Data analysis

**Figure 1.**
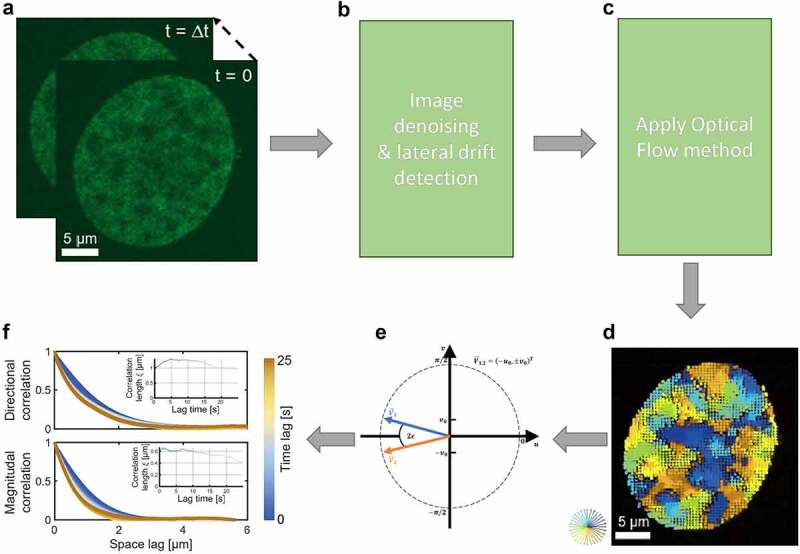
Scheme of DFCC analysis pipeline. **A**) 2D live nucleus imaging of RNA Pol II (RPb1-Dendra2) of U2OS cell line. Images are imaged by spinning disk microscopy with a time interval of Δt (200 ms). **B**) The raw images were pre-processed by testing the lateral drift and applying de-noising step. **C**) Horn–Schunck Optical Flow algorithm was applied to estimate the flow fields for each pixel over the entire nucleus. **D**) displacement fields output of successive two images that are estimated by Optical Flow algorithm. **E**) The vector`s angles were defined on a linear scale. The difference between the angles of the two vectors ${\vec{V}}_{1}$ and ${\vec{V}}_{2}$ in approximately the negative x-direction is 2π−2∈, with ∈ small. Yet, because of the periodicity of 2π, the change can also be defined to 2∈. **F**) The spatial correlation function for the displacement field direction (upper panel) and magnitude (lower panel) is computed over increasing space lags and over accessible time lags (from golden to blue). Whittle–Matérn covariance model for every time lag was applied to calculate the spatial correlation length for both direction and magnitude (insets).

Single fluorescent nuclei were manually cropped (Figure 1a). Preprocessing steps were applied; first, denoising of raw images was applied, and then lateral drift was calculated (Figure 1b). For the denoising step, each frame of the sequence is denoised using non-iterative bilateral filtering to verify the difference in intensity values to preserve the edges of the structures. For our videos, we use a window of 5 pixels, a sigma_space of 5 pixels, and a sigma_intensity of 0.3. Videos with a lateral drift of more than 10 nm were excluded. To calculate the flow (displacement) fields for all analyzed movies, Horn–Schunck Optical Flow algorithm was applied (Figure 1c). Optical Flow is a method that detects the direction and magnitude of motion of labeled molecules. We do this by using median filtering to denoise the flow after each warping step. As a result, it estimates the average displacement of each pixel for stained nuclei within consecutive frames in the nucleus (Figure 1d).

Flow fields were generated on a 100 grid by sampling displacement vectors from a distribution according to the simulated scenario. The magnitude was sampled from a normal distribution with a mean of 0.5 and a standard deviation of 0.05 for flow fields with no correlation (in either magnitude or direction or both). The direction values were drawn at random from a uniform distribution ranging from 0 to 2. A stochastic two-dimensional multifractal random field was used to generate spatially correlated values (for either magnitude or direction or both) [25].

Using circular vector calculation, we calculate the spatial autocorrelation of sets of angles in each flow field to investigate the potential extent of coherent motion. Angles are calculated by taking the arctangent of a vector’s y and x components. Calculated angles are defined on the linear interval, with the positive x-axis chosen arbitrarily as a reference. Angles are calculated by the arctangent of the y- and x-components of a given vector.

(1) $$\gamma =\;\left\{\begin{array}{c} \begin{array}{cc} arctan\left(v/u\right) & if\;u\geq 0,\\ \arctan\left(v/u\right)+\pi & if\;u<0,v\geq 0,\\ arctan\left(v/u\right)-\pi & if\;u<0,v<0, \end{array}\\ \begin{array}{cc} 0 & if\;u>0,v=0,\\ \pi & if\;u<0,v=0,\\ undefined & if\;u=0,\;v=0. \end{array} \end{array}\right.,$$

Angles calculated by equation (1) are defined on the linear interval Precision Oncology Center, Department of Oncology, Lausanne University Hospital, 1005 Lausanne, Switzerland, where the positive x-axis is arbitrarily chosen as a reference. It should be noted that defining angles on a linear interval introduces errors in the spatial correlation of certain angles (Figure 1e). Consider two vectors of unit length pointing roughly in the negative x-direction, with a small positive y-component that can be neglectable. The angle difference between these two vectors is large, but they both point in the same direction. To get around this, the true angle values are expressed in the complex plane using Euler’s formula. In this description, the angle difference is reduced and are small. Circular vector calculation was performed by the use of the Fast Fourier Transform algorithm. Then, the spatial auto-correlation function Precision Oncology Center, Department of Oncology, Lausanne University Hospital, 1005 Lausanne, Switzerland, of a scalar field γ(x,y) of the resulting flow fields’ direction and magnitude for all accessible lag times within flow fields can be calculated as

(2) $$r\left(\Delta x,\Delta y\right)=\;\frac{F^{-1}\left[F\left(\gamma \right)\cdot F^{*}\left(\gamma \right)\right]}{\gamma \gamma },$$

Where $F^{*}\left(\cdot \right)$ and $F^{-1}\left(\cdot \right)$ represent the complex conjugate of the Fourier transformation, and the inverse Fourier transformation. We then calculated the two-dimensional (2D) autocorrelation function for horizontal and vertical space lags, represented by a projection of the 2D correlation function onto one dimension using the space lag $\rho^{2}=\Delta x^{2}+\Delta y^{2}$, which turns the correlation function into a space lag only (figure 1f). Using the Whittle – Matérn covariance function, a regression of these correlation curves over distance was applied.

(3) $$r\left(\rho \right)=\frac{2^{1-\nu }}{\Gamma \left(\nu \right)}{\left(\frac{\rho }{\rho_{c}}\right)}^{\nu }K_{\nu }\left(\frac{\rho }{\rho_{c}}\right)$$

Where Γ (.), (.), ρ, and $\rho_{c}$ represent the gamma function, the modified Bessel function of the second type of order ν, the correlation length, and the smoothness parameter, respectively. After RNA pol II data analysis, we found that there is no significant difference in $\rho_{c}$ across the transcriptional conditions.

## Data Availability

The raw imaging data and analysis files for both that support the findings of this study are openly available in Zenodo Data (https://zenodo.org/record/6998287#.Yv45w0dByUm). Also, the code of analysis is available on GitHub (https://github.com/romanbarth/DFCC).

## Data validation

To validate the extracted parameters by DFCC, DFCC analysis was applied to a series of images of chemically fixed cells. We found that the sensitivity baselines for correlation lengths obtained for both chromatin and RNA pol II R(PB1-Dendra2) in fixed cells were generally well below those observed for live cells and showed no reliance on time delay.(https://doi.org/10.1080/19491034.2022.2088988).
